# Characteristics of the Human Host Have Little Influence on Which Local *Schistosoma mansoni* Populations Are Acquired

**DOI:** 10.1371/journal.pntd.0002572

**Published:** 2013-12-05

**Authors:** Lúcio M. Barbosa, Luciano K. Silva, Eliana A. Reis, Theomira M. Azevedo, Jackson M. Costa, Walter A. Blank, Mitermayer G. Reis, Ronald E. Blanton

**Affiliations:** 1 Gonçalo Moniz Research Center, Oswaldo Cruz Foundation, Salvador, Bahia, Brazil; 2 Bahiana School of Medicine and Public Health, Salvador, Bahia, Brazil; 3 Case Western Reserve University, Center for Global Health and Diseases, Cleveland, Ohio, United States of America; 4 Louis Stokes Cleveland VA Medical Centre, Infectious Diseases Service, Department of Veterans Affairs Medical Centre, Cleveland, Ohio, United States of America; University of Nottingham, United Kingdom

## Abstract

**Background:**

Brazil remains the country in the Americas with the highest prevalence of schistosomiasis. A combination of control efforts and development, however, has sharply reduced its intensity and distribution. The acquisition of specific schistosome populations may be dependent on host characteristics such as sex, age, geography, work, habits and culture. How these and other host characteristics align with parasite subpopulations may guide approaches to improve control.

**Methodology:**

A cohort of more than 90% of the residents in two rural communities in Brazil participated in an epidemiologic survey of demographic, socio-economic and behavioral characteristics. The variables sex, age, intensity of infection, socio-economic index, % lifetime spent on site, previous infection, and trips outside the district were used to group parasites infecting individuals. *Schistosoma mansoni* infection status was determined by examination of stools submitted on 3 different days. The aggregate of eggs collected from the whole stool was used to determine degree of population differentiation from allele frequencies for 15 microsatellites.

**Conclusions/Significance:**

Infection prevalence was 41% for these communities, and the epidemiologic characteristics were similar to many of the endemic areas of Brazil and the world. Parasite population structuring was observed between the two communities (Jost's D 0.046, CI95% 0.042–0.051), although separated by only 8 km and connected by a highway. No structuring was observed when infected individuals were stratified by host's biologic, demographic or epidemiologic characteristics. Those most heavily infected best reflected the communities' overall parasite diversity. The lack of differentiation within villages suggests that individuals are likely to get infected at the same sites or that the same parasite multilocus genotypes can be found at most sites. The geographic structuring between villages and the lack of structuring by age of the host further supports the impression of a population little affected by migration or drift.

## Author Summary

Schistosomiasis is one of the world's most important parasitic infections. Its elimination has proved difficult even in countries such as Brazil where access to treatment is readily available. Infection is the result of human contact with surface water where there are infected snails, so that human biology and habits may bring different individuals in contact with different groups of parasites. Identification of schistosome subpopulations may assist understanding transmission patterns and guide control efforts. We compared microsatellite allele frequencies from all of the infections in 2 small villages and determined that the movement of parasites between them was limited. Individual infections were distinct composites of parasites, but if infected humans were grouped by demographic and epidemiologic characteristics, there was no evidence that specific parasite subpopulations were being selected in these types of hosts. Infections were also not differentiated when stratified by host's age indicating that the populations were stable over time. Since the infection cycle requires human fecal contamination of water, local human behavior can to some degree be inferred from the patterns of schistosome subpopulation distribution.

## Introduction

The transmission of schistosomiasis is influenced by human culture, occupations and demographics among other factors. Also, our group and others have demonstrated that each individual host carries only a portion of the total available parasite genetic variability [1], [2], [3], [4], [5], [6], [7], and thus host-to-host structuring may exist due to each individual's personal characteristics, such as age, sex, social status or residence. These are factors that may bring them into contact with genetically distinct populations of parasites or even influence their susceptibility. While these epidemiologic relationships are usually explored by associating human demographics with infection prevalence or intensity, by using genetic markers we can also determine if these host characteristics are associated with acquiring different parasite subpopulations.

An immediate problem for any such analysis is how to sample the parasite population. Due to the biology and local distribution of the parasite *Schistosoma mansoni*, sampling for genetic analysis is not straightforward. The snail host, where asexual reproduction takes place, lives an average of 3 months, and cercariae collected at one point in time do not represent the whole genetic diversity found in humans [7]. In addition to differences in behavioral factors and biological susceptibility of the human host, the intermittent presence in the snail host increases the potential for differential acquisition of parasite genotypes.

Sexual reproduction takes place in the human host where the adult worms are inaccessibly located in mesenteric veins. A portion of the hundreds of eggs produced daily by worm pairs remains trapped in tissues and will not contribute to the succeeding generation, whereas the majority of these progeny is shed in stool. New individuals enter the host only by infection, a form of migration. Since the adult parasites are long-lived, humans can accumulate a variety of individuals over time. Our approach to the population genetics of *S. mansoni* has been to analyze allele frequencies obtained by extracting DNA from the aggregate of eggs isolated from single stools. In this way the reproducing population of schistosomes from many individuals (e.g., most of the residents of small communities) can be analyzed with a large sample size and a minimum of selection bias.

An important problem for all genetic studies is determining appropriate sample size and avoiding selection bias. The population structure of most organisms is studied by collecting a sample of discrete genotypes and then aggregating or pooling these into allele frequencies for the whole population. This approach is dependent on the quality of the sampling performed. Depending on the organism and the specific population, sample sizes of 30 [8] or hundreds [9], [10] have been deemed necessary to provide an adequate sample. Parasite populations add unique challenges to the problem of sampling since they are not simply structured as discrete organisms scattered or clustered across a landscape. They exist as populations within individual hosts (infrapopulations) as well as the collection of parasites within one host species (component populations) [11]. The latter represents the full genetic potential of which the infrapopulations are each a small sample. For the individual human infection with *S. mansoni*, a typical 200 g stool with a light infection of 40 eggs/g will have a total of 8,000 eggs. The miracidial stage can be hatched from eggs and collected for study. Samples of 10, 20, 30 individual miracidia may be small when diversity is high, and there may be bias for which eggs will hatch into miracidia and which can be collected. Further, the process of hatching and collecting individual parasites limits the number of infected people that can be examined. How to sample, what to sample and how much to sample has never been defined for schistosomes. Our approach to the population genetics of *S. mansoni* has been to analyze allele frequencies obtained by extracting DNA from an aggregate of eggs isolated from the whole stool of infected individuals. In this way the transmitted population of schistosomes from many or even all individuals (in the case of a small community) can be analyzed with a large sample size from many infrapopulations and a minimum of selection bias. Sampling larger numbers of infrapopulations also allows for stratifying hosts for comparisons. Finally, using this approach we have shown that the stool egg population has a similar genetic composition to the adult worm population [12], [13].

In this paper we assessed risk factors for infection and differentiation of parasite infrapopulations by genotyping the aggregate of eggs obtained from infected individuals in two small rural villages. We divided parasites into “component” populations based on host geography as well as host biology, demography and epidemiology. We then estimated differentiation between these groups from their infrapopulation or component population allele frequencies. Although we previously observed structure based on geographic distance between these two nearby communities [3], we found little population structuring within the villages or between hosts. Finally, we explore the implications of these findings for the nature of schistosome populations in rural communities.

## Methods

### Ethics Statement

The Committee on Ethics in Research of the Oswaldo Cruz Foundation of Salvador, Bahia, the Brazilian National Committee on Ethics in Research and the Institutional Review Board for Human Investigation of University Hospitals Case Medical Center, Cleveland, Ohio approved the study design. All subjects provided written informed consent or in the case of minors, consent was obtained from their guardians. All aspects of the study have been conducted according to the principles expressed in the Declaration of Helsinki.

### Study Area

**Figure 1:**
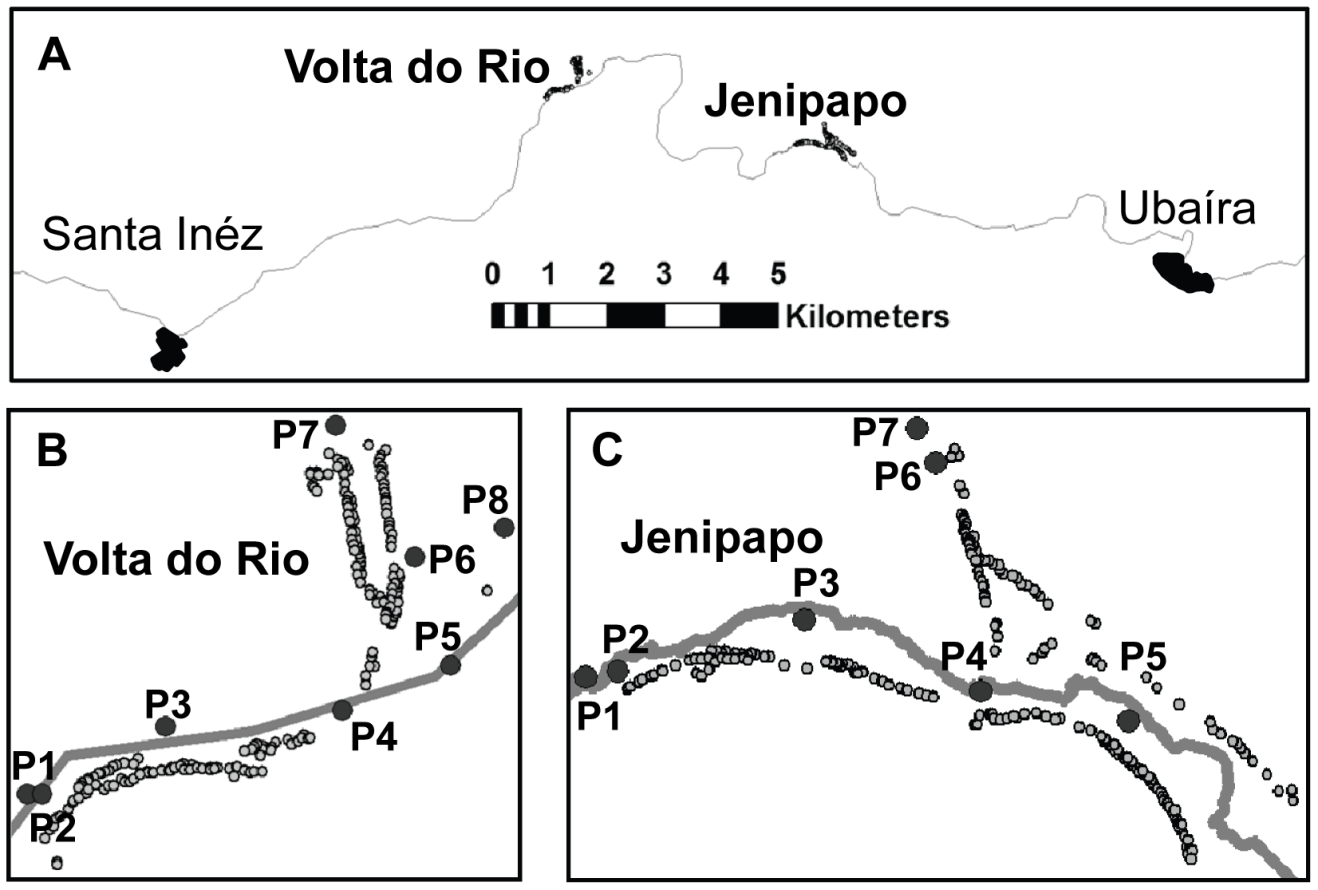
Study areas and water contact sites. A. Map of the Jiquiriçá River Valley between the towns of Santa Inês and Ubaíra. B. Volta do Rio. C. Jenipapo. The Jiquiriçá River is represented by the solid line dividing the communities. Open circles - homes, (P) numbered water contact sites.

Two rural Brazilian communities – Jenipapo (population 482) and Volta do Rio (population 367) – were studied because of their high prevalence of schistosomiasis, their size and their relative isolation. They are administered by the municipality of Ubaíra (roughly equivalent to a county in the USA) and are located in the Jiquiriçá River valley in the state of Bahia. By road they are 270 km SE of the State capitol and principal city, Salvador. Each was at least 12 km from a major town and 8 km distant from each other. Volta do Rio is also divided geographically into an upper and lower section with a 40 m difference in height above the river (Figure 1). The major sources of livelihood are planting cacao, bananas cassava, cattle raising and other animal production. There is a Federal Family Health Program clinic in Jenipapo with a permanent staff consisting of a nurse, dentist and part-time physician. Volta do Rio has a simpler health post that employs only a group of nurses. Jenipapo also has primary and secondary schools attended by all of the nearby small communities, including Volta do Rio.

### Study Design and Protocol

As previously described [3], an epidemiologic and parasitologic survey was conducted for all inhabitants ≥1 year old who agreed to participate. Questions concerning housing, sanitary habits, socio-economic conditions and water contact were asked as part of the epidemiologic survey. For water contact, individuals or guardians for minors <10 years of age were asked if they frequently used any of the 8–9 previously identified major water contact sites and what activities they tended to perform there. The socio-economic evaluation was based on the Criteria for Economic Classification of Brazil (http://www.abep.org/novo/Content.aspx?ContentID=139). These criteria with revisions have been used nationally for more than a decade to characterize the purchasing power of the Brazilian population using possessions (color TV, radio, bathroom, car, washing machine, videocassette/DVD, refrigerator, freezer), services (maid/housekeeper) and degree of education of the head of household. The index places households within 8 categories ranging from minimum monthly wage to 13X minimum monthly wage. The interpretation of these categories is weighted for metropolitan regions of the country including Salvador, Bahia.

Three stool samples each on different days were requested from each resident over a period of 1 week for quantitative examination by the Kato-Katz method. Individuals who tested positive for *S. mansoni* infection were treated with a single oral dose of praziquantel according to Brazilian Ministry of Health guidelines [14]. Those found to have intestinal nematodes were treated with mebendazole.

### Egg Purification

All stools were weighed to the nearest 0.01 g on a digital balance upon arrival in the laboratory. Whole stools from single individuals that were positive for *S. mansoni* were homogenized in a blender containing 200 ml of 2% saline followed by selective sieving [15] through two mesh nylon filter bags (FSI, Michigan City, Indiana, USA) with 300 and 55 µm pore sizes, respectively. The retained material was then sedimented in 2% saline. Since eggs were among the densest elements in the stool [16], the bottom 5 ml of sediment was collected and kept frozen at −20°C until used for DNA isolation.

### DNA Isolation

The 5 ml frozen stool sediment was mixed with 5 ml 2X extraction buffer (50 mM NaCl, 100 mM Tris–HCl, pH 7.5, 10 mM EDTA, 1.0% SDS) and 10 ml H_2_O-saturated and Tris-buffered phenol, pH 7.5. This was followed by two chloroform/Isoamyl extractions [3]. The DNA was then ethanol precipitated and suspended in 10 mM Tris, pH 7.5, 1 mM EDTA. Finally, the sample was treated with cetyl trimethylammonium bromide (CTAB) to remove PCR inhibitors [17].

### Microsatellite Genotyping

To genotype *S. mansoni* eggs, 15 microsatellite markers were used as described previously [2], [3]. For each marker a duplicate PCR reaction using 2 µL of extracted DNA from stool was performed, totaling 30 reactions per sample. PCR products from each sample were combined into groups of three or four markers and processed on an Applied Biosystems 3730xl DNA Analyzer. PeakScanner software (Applied Biosystems, Carlsbad, CA) was used to determine peak heights from which allele frequencies were calculated. Successful PCR reactions were defined as those in which there was at least one peak >500 pixels in the size range expected for a given marker. All peaks less than 100 pixels were excluded. We attempted to genotype all samples, and if multiple samples from the same individual amplified, their mean allele frequency was used. Subsequent population analyses were limited to those samples where a minimum of 12 out of 15 markers genotyped successfully.

### Statistical Analyses

Information collected during the study was double-entered into the program Epi Info version 3.5.3 [18]. Pearson's chi-square and Student's t-test were used to compare categorical and continuous data, respectively, and a p-value of 0.05 was used as the criterion for statistical significance. Multivariable analyses were carried out using logistic or linear regression in SPSS (Version 17). Individuals with missing data were dropped for the analysis of that variable.

For population genetic analyses, allele counts for each sample were calculated by multiplying the allele frequencies at a microsatellite locus by the total egg counts found on the Kato-Katz assay. Infrapopulations were stratified by the host's residence, sex, age, intensity of infection, household, travel history, number and location of water contacts and socio-economic condition. Genetic differentiation between populations was expressed as the index Jost's D [19] calculated using the program SPADE (http://chao.stat.nthu.edu.tw). D is a true differentiation index and does not rely on assumptions of Hardy-Weinberg equilibrium, which do not apply to infrapopulations. After grouping, each pair of infrapopulations can be compared within a group or the combined allele numbers and allele frequencies can be used to form a component population. We make the following differentiation and diversity comparisons:

Di - Jost's D values for pairs of **I**nfrapopulations in the group as defined by host characteristics. The Di indicates how differentiated the parasites infecting individual members of the group are on average from other infections in the group. For any host group a matrix of pairwise Di's is generated, and the mean of this matrix for the different groups was compared.
  Dc - Jost's D for the combined allele numbers of two **C**omponent populations grouped based on shared geography or host characteristics. Differentiation here indicates whether certain host characteristics lead to a preference for selected populations of parasites or vice-versa.
Dic – Jost's D between individual **I**nfrapopulation compared to its geographic **C**omponent population. A single value (Dic) is produced for each individual host that indicates how differentiated his or her infrapopulation of worms/eggs is from the pool of available genotypes. The mean Dic was compared for infrapopulations from humans with and without the selected characteristic.
AE - The effective allele number [20] is a measure of diversity. It was calculated as 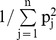 where p_j_ is the frequency of the j^h^ allele for each marker.

Egg counts were recorded as eggs per gram of stool (epg) and log-transformed to approximate a normal distribution for analyses. Arithmetic means were calculated for group Di, Dic and AE. For the Di and the Dic group means were compared by bootstrapped Student's t-test with 1000 resamples, since the distribution of these measures is unknown. There is no standard for effect size for these new types of comparison. For the Dc, we follow the convention used for interpreting *F*_ST_ values [21]. Dc values from 0–0.05 indicate little differentiation; from 0.05–0.15, moderate differentiation; and above 0.15, great differentiation [22]. Changes in D rather than the absolute value below the 0.05 range, however, may still indicate a significant obstacle to gene flow.

## Results

### Study Population Characteristics

The study group consisted of 814 of the 849 (96%) inhabitants residing in the 243 households of the two villages. The mean age was 31.5 years (±22.2), and slightly more women than men were enrolled (53.7%). Most subjects were born in their current municipality (83.7%), and the average percent of lifetime spent in the municipality of Ubaíra was 93.5%. Considering the history of travel outside of the district, 25.3% reported any travel, and a minority (19.5%) of those who traveled reported contact with surface water. There were some differences for the two geographically distinct areas of Volta do Rio (VdR). The percent of those traveling outside of the district was greater for individuals from lower VdR than upper VdR (34.2 vs. 22.2%, p = 0.02), but they remained outside of the area for similar lengths of time (61.92 vs. 57.82 days, p = 0.51). There were significantly more individuals in upper VdR who had at least one family member infected (36.9 vs. 25.2%, p = 0.02).

Most demographic and epidemiologic characteristics were similar for both villages (Table 1), with the exception of the socio-economic index and sanitation. Jenipapo had a somewhat greater purchasing power for (12.0 vs. 10.7, p = 0.017). The mean socio-economic index for the two localities was 11.4±4.3, which corresponds to the second lowest of the 8 income categories used nationwide. A socio-economic index of 11 points translated to a family income of approximately $330/month in 2009. Nearly all homes in both villages have piped water and indoor flush toilets. The 2 most common destinations for these toilets was either a septic tank or the river. Despite a lower socio-economic index, the disposal of human waste was more adequate in VdR than Jenipapo, and upper VdR had better waste disposal than lower VdR. In VdR the Jiquiriçá River is shallow, sluggish and seasonal, while at Jenipapo the Jiquiriçá is joined by a major stream that maintains flow in the river throughout the year. This may explain the different approaches to sanitation. Drinking water in both communities comes from sources several km away from the river.

**Table 1:** Demographic, epidemiologic and infection characteristics of Jenipapo and Volta do Rio.

		Combined	Jenipapo	VdR	*p*	Volta do Rio		*p*
						Upper	Lower	
**Characteristic**	Total	n = 814	n = 461	n = 353		n = 195	n = 158	
**Male Sex**		377 (46.3)	221 (47.9)	156 (44.2)	0.288	92 (47.2)	64 (40.5)	0.209
**Mean age in years**		31.5±22.2	30.6±21.7	32.6±22.7	0.211	32.5±21.8	32.7±23.8	0.922
**Birth place (%)**	Ubaíra	681 (83.7)	391 (84.8)	290 (82.2)	0.309	159 (81.5)	131 (82.9)	0.738
	Other	133 (16.3)	70 (15.2)	63 (17.8)		36 (18.5)	27 (17.1)	
**% Lifetime in Ubaira**		93.5±41.4	93.6±19.9	93.5±58.6	0.982	89.0±23.2	92.5±19.3	0.116
**Travel last year**		204 (25.3)	105 (22.8)	99 (28.7)	0.056	**45 (23.8)**	**54 (34.2)**	**0.033**
**Travel water contact**		39 (19.5)	17 (16.2)	22 (20.6)	0.264	8 (15.1)	14 (26.0)	0.319
**Socio-Economic Index**		**11.4±4.3**	**12.0±4.7**	**10.7±3.8**	**0.017**	10.5±4.2	11.1±3.2	0.390
**Piped Water (%)**		810 (99.4)	-	-	-	-	-	-
**Indoor Toilet**	Yes (%)	791 (97.4)	-	-	-	-	-	-
**Sanitation**	Septic tank	**515 (63.4)**	**200 (43.4)**	**316 (89.3)**	**<0.001**	**187 (95.9)**	**129 (81.6)**	**<0.001**
	River/Open air	**268 (33.0)**	**250 (54.2)**	**20 (7.4)**		**3 (1.5)**	**16 (10.1)**	
**Infection Prevalence (%)**		**335 (41.2)**	**211 (45.8)**	**124 (35.1)**	**0.002**	60 (30.8)	64 (40.5)	0.057
**Infection Intensity (epg** * **)**		60.8	56.6	68.6	0.248	69.2	68.0	0.949
**Past ** ***S. mansoni*** ** infection**	Yes (%)	297 (36.5)	175 (38.0)	122 (34.6)	0.318	68 (34.9)	54 (34.28)	0.891
**Know someone with ** ***S. mansoni***	Yes (%)	252 (31.3)	146 (32.0)	106 (30.0)	0.830	68 (35.4)	38 (24.2)	0.057
**Family member with ** ***S. mansoni***	Yes (%)	252 (34.1)	150 (36.1)	102(31.6)	0.194	**65 (36.9)**	**37 (25.2)**	**0.024**

Categorical variables were compared using Pearson's Chi squared. Means for continuous variables were compared by Students t-test. Significant values are in bold type. Values ± S.D and (%).

^*^ epg - genometric mean of *S. mansoni* eggs per gram of stool. Statistically significant comparisons are in bold type.

### Infection Risk Factors

The prevalence of *S. mansoni* infection was higher in Jenipapo (45.8%) than VdR (35.1%), but the mean intensity of infection was similar (Table 1). The lower limit of detection was 8 epg and the highest mean intensity observed was 3,792 epg. Some 31.3% of residents knew someone with current or past infection with *S. mansoni*, and 34.2% had one or more relatives with schistosomiasis. Two hundred and ninety seven individuals (37%) reported past infection with *S. mansoni*, and 93.6% of those reporting infection also reported being treated, most often with oxamniquine (64.0%). None had been treated with praziquantel, which was newly approved in Brazil for treatment of schistosomiasis at the time of the study. No variables or contact points were correlated with intensity of infection.

**Figure 2:**
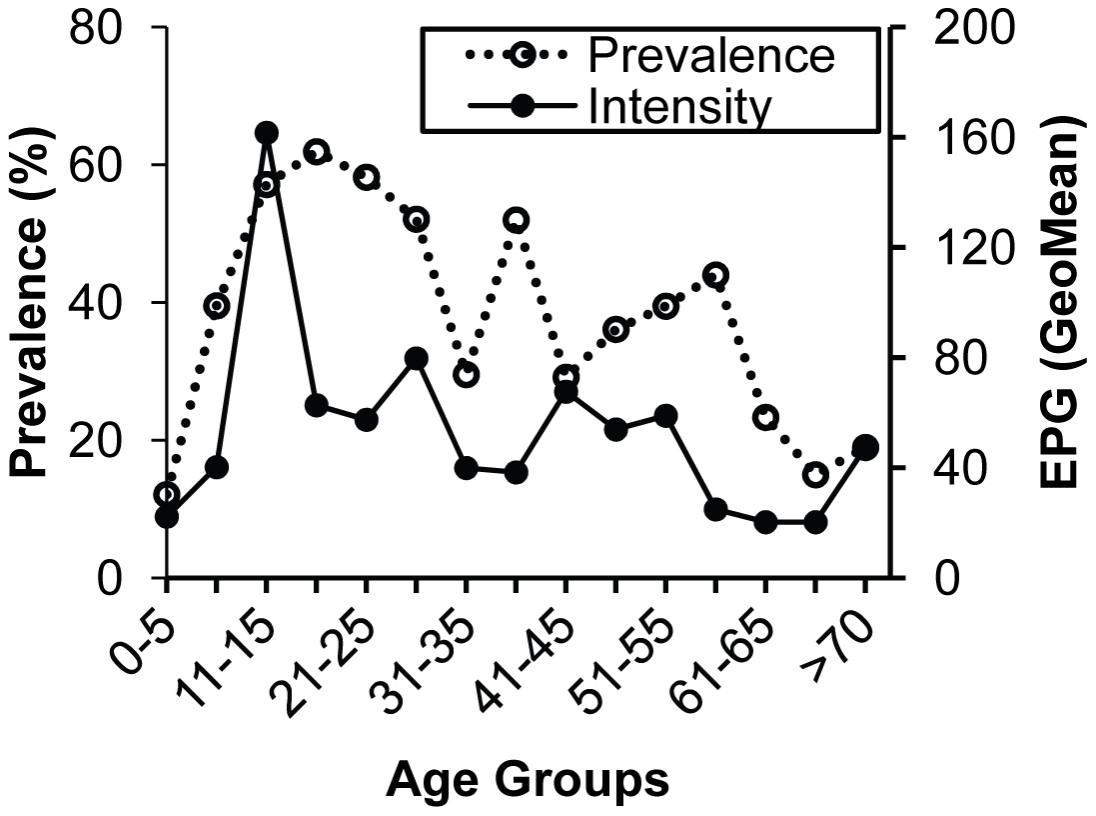
Age-related prevalence and intensity of infection. Prevalence (%) and intensity of infection (geometric mean epg) by age-group. epg - count of *S. mansoni* eggs per gram of stool.

Characteristics that were associated with a higher risk for *S. mansoni* infection were living in Jenipapo, age (2nd, 3rd and 4th decades compared to 1^st^, Figure 2) and male sex (Table 2). Traveling outside of the municipality of Ubaíra in the past year was not associated with an increased risk for infection, but water contact while traveling was (OR of 2.3, p = 0.012) compared to those reporting no contact. A self-reported history of past infection overall had no correlation with risk, but reporting past treatment for *S. mansoni* did (OR 3.07, p = 0.02).

**Table 2:** Risk for *S. mansoni* egg positive stools.

Variable		Total	Positive n (%)	Or (95% CI)	p value
**Age**	1–10	152	42 (27.6)	-	-
	11–20	**175**	**104 (59.4)**	**3.84 (2.41–6.12)**	**<0.001**
	21–30	**138**	**76 (55.1)**	**3.21 (1.97–5.23)**	**<0.001**
	31–40	**94**	**39 (41.5)**	**1.86 (1.08–3.20)**	**0.03**
	41–50	84	27 (32.1)	1.24 (0.70–2.22)	0.470
	51–60	63	26 (41.3)	1.84 (0.99–3.40)	0.052
	>60	108	21 (19.4)	0.63 (0.35–1.15)	0.130
**Sex**	Male	**378**	**192 (50.8)**	**2.12 (1.60–2.82)**	**<0.001**
	Female	**437**	**143 (32.7)**		
**Outside Trips Last Year**	0	602	247 (41.0)	-	
	1–3	148	64 (43.2)	1.10 (0.76–1.58)	0.624
	>4	56	22 (39.3)	0.93 (0.53–1.63)	0.800
**Water contact during trip**	Yes	**39**	**24 (61.5)**	**2.38 (1.18–4.81)**	**0.015**
	No	**176**	**68 (38.6)**		
**Prior ** ***S. mansoni*** ** infection**	Yes	297	128 (43.1)	1.13 (0.85–1.52)	0.393
	No	518	207 (40.0)		
**Treatment for ** ***S. mansoni***	Yes	**278**	**115 (41.4)**	**3.07 (1.13–8.32)**	**0.021**
	No	**19**	**13 (68.4)**		
**Water Contacts**	0	100	18 (17.8)	-	-
	1	**276**	**85 (30.8)**	**1.89 (1.06–3.37)**	**0.032**
	2	**236**	**99 (41.9)**	**2.84 (1.59–5.08)**	**<0.001**
	3	**90**	**56 (62.2)**	**6.07 (3.08–11.94)**	**<0.001**
	4	**56**	**38 (67.9)**	**7.41 (3.43–16.03)**	**<0.001**
	≥5	**56**	**39 (69.6)**	**7.77 (3.56–16.97)**	**<0.001**

Risk for *S. mansoni* infection compared to youngest category for age or to no surface water contact by chi-squared test. CI = confidence interval; OR = odds ratio. Statistically significant comparisons are in bold type.

Eight water contact points in Jenipapo and nine in VdR were identified as those most commonly visited by villagers. The number of visits and nature of activities at each site were asked during the epidemiologic survey. The risk of being infected with *S. mansoni* increased substantially as the individual had contact with an increasing number of sites (Table 2). After adjusting for age and sex, people who reported contact with one point in Jenipapo and two in VdR were significantly more likely to be infected (Table 3). All of these points were common crossings to reach from one side of the river to the highway. A log was used as a temporary bridge at one point each in the two villages. However, at contact point 5 in Jenipapo (Figure 1C) the activity most associated with infection was fishing (OR = 2.96, p = 0.012), which is usually performed while wading in the river. In VdR, at the point not used for crossing the river (P3, Figure 1B) formed a pool, and bathing here was most associated with infection (OR = 3.55, p<0.001). Working and walking at this site were protective (OR = 0.1, p = 0.012 and OR = 0.043, p = 0.036, respectively), while fishing and playing in the water were also associated with a risk for infection (OR = 4.18, p = 0.048 and OR = 4.93, p = 0.026, respectively). The only significant activity associated with those who used the site and were uninfected was collecting water (OR = 5.1, p<0.048).

**Table 3:** Risk at water contact points for *S. mansoni* infection.

Jenipapo (n = 461)					Volta do Rio (n = 352)			
	Infected (%)	Not-Infected	OR (95% CI)	p-value	Infected (%)	Not-Infected	OR (95% CI)	p-value
**Contact point**	n = 211	n = 250			n = 124	n = 230		
1	45 (21.3)	27 (10.8)	1.42 (0.77–2.62)	0.292	**52 (41.9)**	**21 (9.1)**	**2.97 (1.44–6.11)**	**0.001**
2	69 (32.7)	50 (20.0)	1.39 (0.83–2.30)	0.182	48 (38.7)	33 (14.3)	1.84 (0.97–3.49)	0.075
3	31 (14.7)	14 (5.6)	*		**41 (33.1)**	**12 (5.2)**	**3.60 (1.49–8.72)**	**0.004**
4	184 (87.2)	215 (86.0)	*		76 (61.3)	126 (54.8)	*	
5	**110 (52.1)**	**74 (29.6)**	**2.27 (1.52–3.39)**	**<0.001**	15 (12.1)	10 (4.3)	*	
6	49 (23.2)	37 (14.8)	*		17 (13.7)	9 (3.9)	*	
7	11 (5.2)	9 (3.6)	*		37 (29.8)	55 (23.9)	*	
8	-	-	-		8 (6.5)	9 (3.9)	*	
Other	16 (7.6)	13 (5.2)	*		18 (14.5)	17 (7.4)	2.05 (0.90–4.66)	0.091

Reported use of a water contact point (see Figure 1) was entered into a logistic regression model where the dependent variable was *S. mansoni* infection status. The model was controlled for age and sex.

^*^ Indicates contact points that did not enter the model. Statistically significant comparisons are in bold type.

While individuals younger than 15 years old did not report more water contact than those older (p = 0.540), the type of contact may have involved more or longer exposure. Water contact for children tended to involved leisure activities such as walking (p = 0.02), swimming (p<0.001) and playing (p = 0.002) compared to older individuals who contacted water primarily through activities associated with labor, such as working (p = 0.02) and obtaining water (p = 0.05). Fishing was equally frequent between both age groups. Males did report visiting 1.5 times as many water contact points as females (p = 0.001).

### Genetic Differentiation

Our previous study [3] used only samples that were positive by Kato-Katz in all 3 stools (n = 116). For the analysis here we included all samples regardless of the number of stools positive for *S. mansoni*, thus, genotypes from 226 of the 335 infected individuals (67.5%) were included for analysis. Of those genotyped, 51.8% were genotyped for 3 samples, 14.6% for 2 samples and 33.6% for only 1 sample. The differentiation between the two geographic component populations of the two villages (D = 0.046, CI95% 0.042–0.051) was similar to that previously reported [3]. To determine whether related parasites clustered with host characteristics, component populations were formed by grouping infrapopulations based on host epidemiologic characteristics of sex, age, household, economic status, place of birth, frequency of travel, previous infection and number and location of water contacts. Differentiation between these component populations was analyzed for the Di, Dc, Dic and effective allele number.

Di was significantly different for the individual villages and both villages combined when infections were grouped by sex, age, infection intensity and certain water contact sites (Table 4). We also tested similarity of infrapopulations within households. Only 11% and 5% of households in Jenipapo and VdR, respectively, had more than one member infected. The mean Di for household members in Jenipapo was 0.065±0.040 and 0.086±0.047 in VdR compared to 0.095±0.033 and 0.123±0.066 for all those infected in Jenipapo and VdR, respectively. The bootstrapped t-tests for the mean Di of household clusters versus all individuals in the village were significantly smaller (p = 0.004, p = 0.030; Jenipapo and VdR, respectively).

**Table 4:** Subpopulation differentiation and diversity.

	D_i_ (p-value)	D_c_	D_ic_ (p-value)	AE
**Combined**				
Sex (male vs female)	0.125 vs 0.127 (0.105)	0.002	0.061 vs 0.060 (0.961)	3.36 vs 3.31 (0.683)
Age (≤15 vs >15 y/o)	**0.108 vs 0.132 (<0.001)**	0.005	0.053 vs 0.064 (0.069)	3.40 vs 3.31 (0.072)
Intensity of infection (<400 vs >400 epg)	**0.134 vs 0.082 (<0.001)**	0.005	**0.066 vs 0.032 (<0.001)**	**3.40 vs 3.43 (<0.001)**
Socio-economic Index (<11 vs >11)	**0.118 vs 0.129 (<0.001)**	0.004	**0.054 vs 0.067 (0.036)**	3.40 vs 3.29 (0.050)
% lifetime in Ubaira (100% vs <100%)	0.124 vs 0.127 (0.183)	0.013	0.060 vs 0.064 (0.620)	3.35 vs 3.33 (0.555)
Previous Infection (Yes vs No)	**0.124 vs 0.126 (0.028)**	0.003	0.058 vs 0.062 (0.529)	3.36 vs 3.34 (0.661)
Trips outside of the district (Yes vs No)	**0.134 vs 0.122 (<0.001)**	0.007	0.065 vs 0.059 (0.287)	3.32 vs 3.36 (0.358)
**Jenipapo**				
Sex (male vs female)	**0.112 vs 0.101 (<0.001)**	0.003	0.062 vs 0.055 (0.392)	3.35 vs 3.32 (0.653)
Age (≤15 vs >15 y/o)	**0.097 vs 0.112 (<0.001)**	0.006	0.054 vs 0.062 (0.322)	3.28 vs 3.34 (0.228)
Intensity of infection (<400 vs >400 epg)	**0.116 vs 0.054 (<0.001)**	0.006	**0.065 vs 0.028 (<0.001)**	**3.32 vs 3.40 (<0.001)**
Socio-economic Index (<11 vs >11)	**0.098 vs 0.115 (<0.001)**	0.007	0.054 vs 0.065 (0.176)	**3.35 vs 3.31 (0.042)**
% lifetime in Ubaira (100% vs <100%)	0.107 vs 0.097 (0.111)	0.008	0.059 vs 0.061 (0.848)	3.36 vs 3.32 (0.520)
Previous Infection (Yes vs No)	**0.102 vs 0.110 (<0.001)**	0.002	0.056 vs 0.062 (0.435)	3.39 vs 3.33 (0.195)
Trips outside of the district (Yes vs No)	0.111 vs 0.105 (0.016)	0.011	0.062 vs 0.058 (0.610)	3.33 vs 3.37 (0.456)
Contact point 5 (Yes)	**0.092 vs 0.124 (<0.001)**	0.006	**0.050 vs 0.070 (0.014)**	3.39 vs 3.32 (0.188)
**Volta do Rio**				
Sex (male vs female)	**0.108 vs 0.128 (<0.001)**	0.007	0.059 vs 0.070 (0.191)	3.37 vs 3.19 (0.136)
Age (≤15 vs >15 y/o)	**0.091 vs 0.121 (<0.001)**	0.009	**0.049 vs 0.067 (0.035)**	3.42 vs 3.27 (0.216)
Intensity of infection (<400 vs >400 epg)	**0.124 vs 0.079 (<0.001)**	0.008	**0.068 vs 0.039 (<0.001)**	**3.26 vs 3.48 (0.016)**
Socio-economic Index (<11 vs >11)	**0.104 vs 0.128 (<0.001)**	0.005	0.055 vs 0.071 (0.074)	3.32 vs 3.43 (0.133)
% lifetime in Ubaira (100% vs <100%)	0.115 vs 0.116 (0.870)	0.020	0.063 vs 0.065 (0.814)	3.34 vs 3.34 (0.974)
Previous Infection (Yes vs No)	0.116 vs 0.116 (0.970)	0.018	0.063 vs 0.063 (0.968)	3.31 vs 3.37 (0.319)
Trips outside of the district (Yes vs No)	**0.129 vs 0.112 (<0.001)**	0.013	0.071 vs 0.060 (0.301)	3.32 vs 3.35 (0.619)
Contact point 1 (Yes)	**0.107 vs 0.126 (<0.001)**	0.006	0.057 vs 0.069 (0.176)	3.34 vs 3.26 (0.970)
Contact point 3 (Yes)	**0.102 vs 0.130 (<0.001)**	0.008	**0.049 vs 0.073 (0.005)**	3.37 vs 3.25 (0.320)

Di - pairwise D for all members of the group. Student's t-test was used to compare group means. Di and Dic were compared by ANOVA Other variables were trips outside of the region, co-infection with other helminths, all water contact points, number of water contacts visited, a history of past infections, socio-economic index. The Dc was estimated using the program Spade (http://chao.stat.nthu.edu.tw). Statistically significant comparisons for Dic are in bold type.

The Dc indicates that the composition of the populations based on host characteristics differ little in their genetic composition. The Dic was significant for age overall, but this was mainly due to a difference in VdR where children ≤15 acquired parasites that were more genetically differentiated from the whole community of parasites than those infecting adults. Overall and in both communities, infrapopulations from heavy infections were less differentiated from the community's component population than lighter ones.

The AE was only significantly different for intensity of infection for the two villages combined as well as separately. This is a measure of diversity, and higher intensity infections averaged higher effective allele numbers. AE was also associated with the socio-economic index in Jenipapo.

Since different age groups may be exposed to different subpopulations of *S. mansoni*, we further stratified age into 4 groups: 0–7, 8–15, 16–40 and >40. We found that the youngest age group gave the highest Dc in pairwise comparisons and the highest mean Dic of any group, but this age group was also the smallest (n = 12), had the lowest prevalence and the lowest intensity of infection. When 12 individuals with similar intensities of infection (sample sizes) were compared from each age group, these differences resolved.

## Discussion

The communities of Jenipapo and Volta do Rio are typical of the region in their level of development and access to sanitation. They also are similar to many other areas endemic for schistosomiasis in their age-specific prevalence and intensity of infection [23]. Differences in the prevalence of infection between these otherwise similar communities may be due to differences in how human waste is handled. In part these choices may be the result of the presence of constant flow in the river in Jenipapo and seasonal flow in VdR. Consistent with this, upper VdR which is much further from the river had higher use of septic tanks and fewer homes reporting using the river. In Brazil, economic development and control efforts using education and the drug oxamniquine (used prior to praziquantel) have greatly reduced the amount of hepatosplenic disease, but the infection prevalence in many areas has not changed. In these two villages, the current prevalence when based on a single stool examination is no different from the 15–20% prevalence observed for the state of Bahia in the 1950's [24], [25] and at the start of control programs in 1976 [24], [26], [27]. When multiple stool samples are examined, the true prevalence of infection is even two to threefold higher.

Some common risk factors found in other communities can be identified in this study. Age between 10 and 20 was associated with the highest prevalence and intensity. In Brazil, male sex is associated with increased risk [28], but in other parts of the world infection can be more prevalent in females [29]. This difference is likely due to differing sexual roles in work, play and ultimately water contact. Water contact is an essential step in transmission of schistosomiasis. While this variable would seem to be a strong risk factor with high correlation with infection, it has been difficult to measure and then associate with intensity [30]. Even when water contact is directly observed [29], the frequency of contacts is not always predictive. Questionnaires have been the simplest and least expensive way to assess risk factors. In Brazil, questionnaires have been shown to produce reliable responses that correlate with risk of infection [31], [32], but even here there can be significant place-to-place variation [33] requiring questions tailored to the specific location. Water contact has been solicited in multiple ways in terms of location, type of activity, time of contact and percent body exposure. We asked only which sites were visited and what activities were commonly performed there. The questionnaire was administered prior to all stool examinations and thus not biased by knowledge of the infection status of respondents. We found that simply counting the number of sites visited was most associated with prevalence of infection. Further, while travel away from the area was not associated with infection, self-reported travel combined with surface water contact was associated. These associations tend to validate the responses given by the residents.

In addition to risk for prevalence and intensity of infection, we sought to identify risk factors for acquiring specific parasite populations. The moderate differentiation between infrapopulations indicates that each individual collects a limited portion of the total genetic variability from the component population. This non-homogeneous distribution together with differences in water contact, occupation, habits, sex, years of exposure, etc are all reasons for structuring of the parasite population within different demographic categories of the human host. No population structuring, however, was observed. In another human population with a different intensity of transmission or different economic, cultural or geographic organization the distribution of parasites might be different.

Geographic structuring showed that over a short distance schistosome gene flow is limited in this region (Dc Jenipapo/VdR = 0.046). By contrast, within the two villages, when we assessed differentiation based on individual water contact sites, we found no difference in Dc among the sites or for number of sites visited. In VdR in particular, where there is a significant geographic difference in the height of the two parts of the city relative to the Jiquiriçá River and a highway between them, we were unable to demonstrate geographic differentiation. This indicates that, within the resolution of our methodology, local gene flow is high within the villages, but not between them. Individuals tend to be infected at the same sites or the same parasite multilocus genotypes can be found at most sites. They do not tend to contaminate the waters in nearby villages, and the school sanitation system (serving children from both villages) is unlikely to contribute to the local parasite population.

The marked difference in age-specific prevalence, intensity and perhaps increased exposure during water contact suggest that children are likely to be more exposed than adults to the current component population present in the resident snails. However, the lack of differentiation by age suggests that current and past populations are largely undifferentiated, and that over at least the last 5 years (the 95% CI for parasite life-span is 5.7–10.5 years [34]) there has not been a large degree of migration or selection, also supported by the geographic structuring between the villages.

The amount of differentiation within the groups of infrapopulations (Di) defined by host characteristics was often significantly different for multiple host factors, but we have no basis for comparison to say if this is biologically meaningful. By contrast the index Dic was significantly different for only intensity of infection in both villages. The socio-economic index and age are variably associated, but these may be secondarily related to intensity. The Dic is a measure of how differentiated an individual infrapopulation is from the whole adult worm/egg parasite community. It serves as a useful measure of how effectively individual hosts within a group sample the component population. The higher the intensity of infection, the more samples are present, which results in a better representation of the component population. This will reduce D for the infrapopulation relative to the component population. For the mean effective allele number, a measure of diversity, only intensity of infection (<400 or >400 epg) showed significant difference for both villages. This is consistent with expectation. In this area, the sampling of the most heavily infected, who are usually between 7 and 15 years of age, might be the best way of estimating the composition of the component population without sampling everyone. This limited sample would still lack precision, and age alone was not significantly associated with the Dc or Dic for Jenipapo.

An important issue for these conclusions is the sensitivity of the methods employed for differentiating parasite subpopulations. We do know that our approach is sensitive enough to differentiate the component population for the two villages, and that in the laboratory, different laboratory-maintained *S. mansoni* populations from the same laboratory and different lots of parasites from the same life cycle can be distinguished [12]. We were able to genotype at least 1 sample from 67% of those infected. Since we have shown infrapopulation allele frequencies are stable over at least the span of a week, obtaining a single stool is unlikely to be a source of error. Most of those we were unable to genotype had low egg counts and low DNA concentrations [3]. Most of the cryptic infections we failed to detect are also likely to have been of low intensity. They were, therefore, less likely to contribute significantly to the genetic composition of their component populations. The relative relationship of the genetic composition of eggs to adult worms is unknown in natural infections, but in laboratory infections in mice, allele frequencies between these two stages were very similar [12].

There is no one approach that address all problems in population genetics, but the approach taken here is well suited to measure differentiation, since it allows for many large samples. Certain population genetic indices, such as the FIS, cannot be well estimated from aggregated data, however. In addition, we are unable to identify null alleles. This should not affect estimates of differentiation for populations in which the rate of null alleles is likely to be similar.

Attempts to control *S. mansoni* infection in Brazil were successful in decreasing intensity of infection, and therefore, morbidity and mortality of the disease, but the infection has far from disappeared. An understanding of the dynamics of transmission and the distribution of the parasite at the population level can contribute to planning control measures. We show that there is little population sub-structure by host characteristics to influence how praziquantel therapy should be distributed. There are no special reservoirs of distinct parasite populations within the community, and much of transmission is local with good evidence for a barrier to gene flow with a nearby community. Future studies will examine how applicable the patterns seen in these communities are to others in Brazil and elsewhere. Until elimination has been achieved, surveillance and treatment will need to be continued and improvements in sanitation advanced.

## Supporting Information

Checklist S1 STROBE checklist.
(PDF)
